# Dihydroxyacetone: An Updated Insight into an Important Bioproduct

**DOI:** 10.1002/open.201700201

**Published:** 2018-03-06

**Authors:** Rosaria Ciriminna, Alexandra Fidalgo, Laura M. Ilharco, Mario Pagliaro

**Affiliations:** ^1^ Istituto per lo Studio dei Materiali Nanostrutturati, CNR via U. La Malfa 153 90146 Palermo Italy; ^2^ Centro de Química-Física Molecular and IN-Institute of Nanoscience and Nanotechnology Instituto Superior Técnico, Universidade de Lisboa Av. Rovisco Pais 1 1049-001 Lisboa Portugal

**Keywords:** bioeconomy, bioproduct, dihydroxyacetone (DHA), glycerol, self-tanning products

## Abstract

Currently obtained from glycerol through microbial fermentation, the demand of 1,3‐dihydroxyacetone (DHA) has significantly grown during the course of the last decade, driven by the consumer passion for a tan and increasing awareness of UV photodamage to the skin caused by prolonged exposure to the sun. We provide an updated bioeconomy perspective into a valued bioproduct (DHA), whose supply and production from glycerol, we argue in this study, will rapidly expand and diversify, with important global health benefits.

## 1 Introduction

**Scheme 1:**
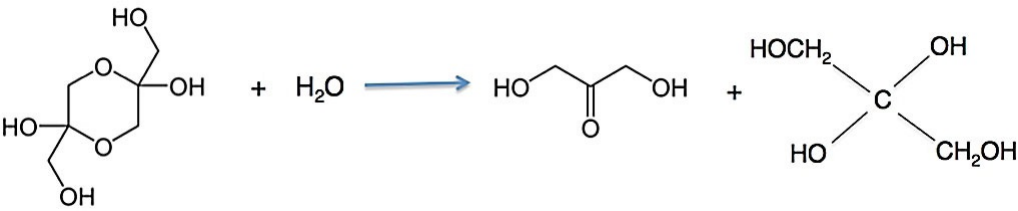
Dimeric DHA in the solid state dissociates into a mixture of free carbonyl and hydrated monomers upon dissolution.

Commercially obtained from glycerol through microbial fermentation, over the acetic acid bacteria, 1,3‐dihydroxyacetone (DHA; 1,3‐dihydroxy‐2‐propanone) is the simplest ketone form of sugars (ketoses) and an important intermediate in carbohydrate metabolism in higher plants and animals formed during glycolysis.^1^ In the solid‐state, DHA exists as a dimer with a dioxan structure, which, upon dissolution, readily dissociates into a mixture of free carbonyl and hydrated monomers (Scheme 1).^2^

In a typical Maillard reaction (the reaction of sugars with amino acids, ubiquitous in cooking and, thus, in daily life),^3^ DHA reacts with the protein keratin on the skin surface, producing pigments called melanoidins, polymeric compounds that are linked by lysine chains to the proteins of the stratum corneum.^4^

Its browning effects, exploited also to mask the effects of vitiligo (treatment with a 6 % DHA cream, leading to 90 % satisfaction of vitiligo patients),^5^ were discovered by accident in the 1930s: attempts to treat diabetes patients with oral doses of DHA resulted in a deep yellow coloring of gums.^6^ The first self‐tanning lotion is said to have been commercialized in 1945 in California,^6^ though most reports agree that sunless tanning products debuted on the US cosmetic market in 1959.^7^

In any case, in the 1950s, Wittgenstein rediscovered the skin browning caused by DHA while studying the effect of large oral doses of DHA in children with glycogen storage disease. The skin of the children that casually and accidentally came into contact with DHA in solution turned brown, whereas textiles did not.^8^ Wittgenstein and Berry concluded their 1960 “artificial tan” study^9^ by reporting findings on the mechanism of skin staining with DHA, stating that it “appears to proceed through combination with free amino groups in skin proteins, and particularly by combination of DHA with the free guanido group in arginine”.^9^

Fifty five years later (in 2015), driven by consumer demand for a fashionable tan throughout the whole year and by concomitant increasing awareness of UV photodamage hazards,^10^ one of the leading self‐tanning product manufacturers sold out a year's worth of stock of its in‐shower tanning lotion in only one day.^11^ Already in 2011, 41 % of women in the UK were reported to use self‐tanning products.^12^ In this study, we provide an updated bioeconomy perspective into a valued bioproduct (DHA), whose supply and production from readily available glycerol, we argue, will rapidly expand and diversify.

## 2 Quality and Health Aspects

DHA has been approved across the world for use in self‐tanning products for several decades. To quote an industry's practitioner “no other substance, so far, has been capable to provide more satisfactory or more lasting results”.^6^ In the EU, for example, the Scientific Committee on Consumer Safety evaluated the safety of DHA as a self‐tanning ingredient in cosmetic formulations, in 2010, and concluded that the use of DHA in skin formulations at concentrations up to 10 % will not pose a risk to the health of the consumer.^13^ Several medical associations recommend it as a safer alternative to UV radiation from the sun or from hazardous tanning beds (“we don't call those UV tanning booths; we call them tanning coffins”).^14^ Exposure to UV radiation and, in particular, to indoor tanning beds can cause skin cancer, skin burns, and premature skin aging. Current misconceptions among the general public about the established risks of DHA‐containing sunless tanning products, as well as guidelines for their proper use, have been reviewed lately.^15^

Sunless tanning products containing DHA produce, within a few hours, a relatively long‐lasting tan (from 3 to 10 days, depending on the formulation), without the risks of photodamage. As mentioned above, this reaction is limited to the stratum corneum (the outer layer of human skin comprised of dead cells), and in vitro skin absorption studies have found no significant systemic absorption of DHA when applied topically to the skin.^16^ On the other hand, the Maillard reaction between DHA and amino acids generates reactive oxygen species (ROS), namely highly reactive free radicals,^17^ that may attack the cell structures and degrade collagen and elastin fibers, promoting premature skin aging and wrinkle formation. The process is accelerated under sun radiation, with more than 180 % additional radicals generated during sun exposure with respect to untreated skin, thus requiring short or no sun exposure when self‐tanners are used.^18^ It is also relevant here to notice how, when applied on the skin, a typical DHA‐based cream attenuates the sunlight‐induced formation of vitamin D.^19^

Owing to the formation of ROS, it is perhaps not surprising that DHA induces DNA damage.^20^ More recently, scholars in the US confirmed and expanded these results, showing that exposing viable cells to DHA significantly alters the cell microenvironment, promoting the induction of cell death, in particular through internal exposures from inhalation, absorption into mucous membranes, or through broken skin.^21^ The team concluded that more work is necessary to understand the complex metabolic events induced by exposure to DHA.

Meanwhile, as DHA continues to be approved as a self‐tanning ingredient, this is a clear case in which chemical innovation aimed at product quality improvement, concomitantly leads to minimized potential health and safety risks, while still avoiding the UV‐induced skin photodamage of conventional tanning.

Conventional self‐tanning formulations use relatively high levels of DHA (up to 15 %), causing unnatural orange tones, smell due to the Maillard reaction (a burnt biscuit stench), and uneven deposition of color and skin dryness. Successful efforts devoted to solve these issues and improve the artificial tanning process include: increased stability (avoiding incompatible ingredients in the final formulation, whose pH must be below 5 for DHA stability); reduced damage from free radicals (through the addition of powerful natural antioxidants); enhanced tan, lasting longer and providing a more pleasing color tone (by adding erythrulose, another keto‐sugar occurring in red, to the formulation).

Further improvements originate from purer DHA made available by suppliers, preferably in powder form (the degradation of DHA in this form is negligible when stored at room temperature for one year; whereas a 10 % DHA aqueous solution stored at 40 °C for 6 months shows a loss of approximately 25 % of the active ingredient).^22^

A non‐exhaustive Review^23^ of recent progress started in 2011, when research chemists at a large chemical company introduced a new formulation, in which DHA was added in a low amount (from a concentration of ca. 0.01 % to ca. 0.9 %) to a topical cosmetic base comprising moisturizers, vitamins, botanicals, oils, and sunscreen agents. This was formulated as an emulsion with the aid of an emulsifier suitable for topical use on skin, such as cetearyl glucoside, which strengthens the lipid structure within the skin, establishing a barrier to moisture loss.^23^ Other examples of similar commercial formulations include hyaluronic acid (to keep the skin hydrated), hemp seed oil extract containing all 21 amino acids for a healthy skin, black tea, and Aloe Vera antioxidants with soothing properties.^24^ Furthermore, some of the DHA added to the formulation is microencapsulated, so as to provide a long‐lasting tan through the slow release of the entrapped molecules as well as to assure a longer shelf life.

Another new formulation consists of tailor‐made tanning products based on liquid concentrates, blending DHA with raspberry oil and Aloe Vera, which users can combine with their moisturizer to create multi‐tasking skincare products, with even sleep‐mask tan formulations to replace conventional nightly skincare masks.^25^ The list concludes with a self‐tanning product that includes a blend of EcoCert‐certified DHA and erythrulose with cardamom seed oil and five aromatic teas, so as to avoid the use of any phthalate‐containing fragrance to mask the unpleasant smell that often characterizes sunless tanners: natural pigments beet root, blue green algae, caramel, and cocoa powder.^26^

In general, the new formulations make the skin appear more moisturized, and the color more natural and more radiant over an extended period of time, thereby solving the old orange‐looking and odor issues typical of former self‐tanning compositions.^23^ With today's advanced formulations, no professional application of sunless tanning products or complex skin preparation steps are required, with products ranging from portable lotions to post‐shower formulations.

## 3 Market and Production

The global self‐tanning product market was recently forecast to generate $1.011 million revenue in 2017.^27^ For comparison, global revenue in 2014 was around $775 million.^27^ In the US, from 2011 to 2016, the market grew by 27 % (from $135 million to $171 million), and by another 19 % in 2017 alone.^11^

In 2013, Dobos emphasized how improved self‐tanning products that were capable of gradually providing a more natural color had become so successful that consumer demand resulted in product shortages, causing “bidding wars”^7^ on a well‐known online shop. By the same token, with several million cases of non‐melanoma skin cancer treated every year across the world, the use of self‐tanning products by increasingly aware consumers, willing to avoid the hazards of long‐term sun exposure, will only increase. This means that there is a need to expand and renew the conventional production of DHA. Accordingly, by analyzing the global DHA industry, a market research company recently concluded that the “development of technology is the key”.^28^ The industrial production of DHA involves the biotransformation of pure glycerol in aqueous solution through the free cells of the acetic acid bacteria *Gluconobacter oxydans* in a reaction catalyzed by glycerol dehydrogenase.^29^ This microbial process typically requires reaction times of up to 70 h, with a maximum yield of 40 %, leading to a high production cost.

Concomitant to market expansion, numerous attempts have been devoted to improving the conventional method. Suffice it to mention here, the conversion of glycerol to DHA has been mediated by the glycerol dehydrogenase enzyme encapsulated in magnetic mesoporous silica, with the solid catalyst being easily separated from the reaction mixture (by using a simple magnet) and recycled in seven consecutive cycles.^30^ The use of a raw biodiesel glycerol by‐product in place of pure glycerol has also been converted over an alginate‐immobilized biocatalyst (cell extract), resulting in a similar concentration of DHA (8.7 g L^−1^) in only half the time.^31^

Figures on the DHA market volume greatly differ. A 2010 scientific article from Japanese scholars^32^ suggests that the annual production of DHA would amount to 2000 tons. Indeed, as a chemical imported in the EU market in quantities of 1000–10 000 tons per year, DHA appears in the European Chemicals Agency register, in which two new suppliers registered as of late 2017.^33^

Similarly, publicly available prices for DHA differ greatly. Scholars in South Korea in 2015 reported a price of $150 kg^−1^,^[30 ]^emphasizing how attractive this made the conversion of glycerol to DHA, given the low price of glycerol ($0.35 kg^−1^). Glycerol derived in huge amounts from biodiesel manufacturing has become an inexpensive platform chemical.^34^ All of these arguments anticipate the forthcoming development and commercialization of chemical catalysis affording high yields of DHA, especially in view of low capital and operational costs.

A first major advance was reported by Koper and co‐workers in 2012, as they obtained 100 % selectivity in the electro‐oxidation of glycerol to DHA at the surface of a PtBi/C electrode modified with bismuth.^35^ No chemical oxidant was required besides the electric current.

**Figure 1:**
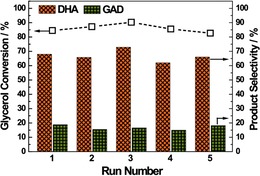
Stability test of the PtSb/C electrode for glycerol oxidation [reaction conditions: 0.1 m glycerol, 0.797 V, 60 °C, 10 h] in a mixture of 0.1 m glycerol and 0.5 m H_2_SO_4_ at a constant potential of 0.797 V (vs. SHE). Reproduced from Ref. 36 with permission. Copyright (2016) Royal Society of Chemistry.

Though less selective, mediating the electrocatalytic oxidation of glycerol to DHA in 61.4 % yield (and about 15 % yield of glyceraldehyde) at a glycerol conversion of 90.3 % under moderate voltage (0.797 V vs. SHE, standard hydrogen electrode), the PtSb/C electrocatalytic electrode was shown, by scholars in South Korea in 2016, to be highly stable. Reused for five consecutive reaction cycles, after washing several times with water and drying in an oven at 343 K for 2 h prior to reuse, the electrocatalytic electrode retained its high original activity and selectivity, affording glycerol conversion and selectivity values with variations of only around 5 % (Figure 1).^36^

Another major advance was reported by Xu and co‐workers in 2013,^37^ when they showed how to heterogeneously oxidize glycerol to DHA by using O_2_ as the primary oxidant in water, at room temperature and atmospheric pressure, over a nanostructured flower‐like Bi_2_WO_6_ photocatalyst, under visible‐light irradiation. Though carried out in a diluted 0.067 m solution, the new photocatalytic process afforded unprecedented yield (87 %) and selectivity (91 %) values. The process was even improved 2 years later with the sol‐gel encapsulation of the photocatalyst, which resulted in a threefold enhanced reaction rate, while retaining the striking selectivity to DHA.^38^ Almost concomitantly, a similar technology was developed in Germany, based on a heterogeneously photocatalyzed process under normal conditions (room temperature, ambient pressure) using α‐Bi_2_O_3_/Pt powder as the visible‐light photocatalyst from a concentrated aqueous solution of glycerol (1:1 glycerol/water by volume).^39^ Remarkably, a company in Germany already offers the technology for transfer to industry.^40^

## 4 Outlook and Conclusions

Since the 1920s, when tanning was made trendy by Coco Chanel,^41^ a tan has become highly fashionable across the world. Based on the use of new bioproducts and microencapsulation technology, significant progress in self‐tanning products using DHA as the active ingredient has been able to solve the main issues of former self‐tan formulations. As these new formulations penetrate the market, the negative reputation of self‐tanning products will become obsolete, and self‐tan utilization may become as commonplace as the use of lipsticks and other makeup. Advanced producers even offer online applications (*Tan Mirror*), allowing users to take a photograph and see how they will look after application. Driven by product quality, such progress has afforded benefits to health and safety issues, as new formulations comprise much lower amounts of DHA, containing powerful natural antioxidants that are capable to quench the ROS free radicals generated on the skin upon the application. This resulted in a net positive contribution to health by avoiding prolonged exposure to the UV radiation, both under the sun and in tanning booths and beds. Globally, this has led to a booming demand of high‐purity and possibly even certified DHA. Although market figures are generally not publicly available, we assume that the current annual DHA production has more than doubled from the 2 000 tons produced in 2009/2010. Along with advances in conventional DHA manufacturing through glycerol microbial oxidation, the aforementioned high demand and sustained high prices open the route to the introduction of industrial DHA catalytic syntheses. In particular, we argue, in this study, that the oxidant‐free electrocatalytic oxidation of glycerol over new and highly stable electrodes^37^ and the aerobic photooxidation driven by solar light^35^ are ideally suited for industrialization. This article offers an updated bioeconomy perspective that will be useful to researchers and industry practitioners in accelerating the progress of DHA manufacturing and the production of self‐tan formulations capable of deploying the full potential of this unique molecule.

## Conflict of interest

*The authors declare no conflict of interest*.
